# Functionalization of gadolinium metallofullerenes for detecting atherosclerotic plaque lesions by cardiovascular magnetic resonance

**DOI:** 10.1186/1532-429X-15-7

**Published:** 2013-01-16

**Authors:** Anthony Dellinger, John Olson, Kerry Link, Stephen Vance, Marinella G Sandros, Jijin Yang, Zhiguo Zhou, Christopher L Kepley

**Affiliations:** 1 Luna Innovations Incorporated, Luna nanoWorks Division, 521 Bridge St, Danville, VA, 24541, USA; 2 Joint School of Nanoscience and Nanoengineering, 2907 E Lee St, Greensboro, NC, 27401, USA; 3 Center for Biomolecular Imaging, Wake Forest University, 1 Medical Center Blvd, Winston Salem, NC, 27157, USA; 4 Carl Zeiss Microscopy, LLC, One Zeiss Drive, Thornwood, NY, 10594, USA

**Keywords:** Atherosclerosis, Magnetic resonance imaging, Metallofullerenes, Contrast agent, Macrophage, CD36

## Abstract

**Background:**

The hallmark of atherosclerosis is the accumulation of plaque in vessel walls. This process is initiated when monocytic cells differentiate into macrophage foam cells under conditions with high levels of atherogenic lipoproteins. Vulnerable plaque can dislodge, enter the blood stream, and result in acute myocardial infarction and stroke. Imaging techniques such as cardiovascular magnetic resonance (CMR) provides one strategy to identify patients with plaque accumulation.

**Methods:**

We synthesized an atherosclerotic-targeting contrast agent (ATCA) in which gadolinium (Gd)-containing endohedrals were functionalized and formulated into liposomes with CD36 ligands intercalated into the lipid bilayer. *In vitro* assays were used to assess the specificity of the ATCA for foam cells. The ability of ATCA to detect atherosclerotic plaque lesions *in vivo* was assessed using CMR.

**Results:**

The ATCA was able to detect scavenger receptor (CD36)-expressing foam cells *in vitro* and were specifically internalized via the CD36 receptor as determined by focused ion beam/scanning electron microscopy (FIB-SEM) and Western blotting analysis of CD36 receptor-specific signaling pathways. The ATCA exhibited time-dependent accumulation in atherosclerotic plaque lesions of ApoE −/− mice as determined using CMR. No ATCA accumulation was observed in vessels of wild type (C57/b6) controls. Non-targeted control compounds, without the plaque-targeting moieties, were not taken up by foam cells *in vitro* and did not bind plaque *in vivo*. Importantly, the ATCA injection was well tolerated, did not demonstrate toxicity *in vitro* or *in vivo*, and no accumulation was observed in the major organs.

**Conclusions:**

The ATCA is specifically internalized by CD36 receptors on atherosclerotic plaque providing enhanced visualization of lesions under physiological conditions. These ATCA may provide new tools for physicians to non-invasively detect atherosclerotic disease.

## Background

Atherosclerotic cardiovascular disease results in close to 20 million deaths annually. A hallmark of the disease is the accumulation of plaque lesions in blood vessel walls which can rupture and result in acute myocardial infarction and stroke. Clearly better diagnostic tools are needed to identify incipient disease, monitor disease progression, and pinpoint factors that predict catastrophic ruptures.

At present, it is difficult for physicians to specifically detect and quantify plaque lesion buildup in vessel walls. Cardiovascular magnetic resonance (CMR) is one of several techniques being investigated to identify plaque burden in patients so that interventions can be conducted before rupture occurs [1,2]. We have used novel gadolinium (Gd)-containing C_80_ endohedrals (Trimetaspheres™, TMS, Gd_3_N@C_80_) [3] as a platform to develop new atherosclerotic targeting contrast agents (ATCA) for CMR. The TMS-based molecules have 25 fold increased relaxivity compared to other contrast agents, reduced risk of metal toxicity, and can be customized to address issues surrounding solubility, specificity, etc. [4]. TMS were functionalized with highly specific ligands for the scavenger receptor CD36 found on the surface of macrophage foam cells in plaque lesions [5]. It is demonstrated that ATCA specifically bind to and are taken up within foam cells *in vitro* and are able to detect lesions in plaque-susceptible mice *in vivo* (Apolipoprotein E deficient mice [ApoE −/−]). Thus, TMS can be targeted to specific biomarkers in sufficient quantities to provide enhanced imaging of atherosclerotic plaque under physiological conditions. These results suggest that the ATCA may be a new tool for detecting atherosclerotic plaque.

## Methods

### ATCA synthesis, functionalization, and characterization

The method to synthesize the TMS uses an electric-arc process to encapsulate Gd within a carbonaceous cage, C_80_[3], which was used as the starting material for the ATCA. The molecular weight of TMS (1,446 Da) was determined using matrix-assisted laser desorption/ionization (MALDI) with a time-of-flight (TOF) mass spectrometer. Elemental analysis determined that TMS contains 10.15 ± 0.25 mM Gd which approximates the value estimated from the molecular weight. No free Gd (Gd outside the carbon cage) was detected using an Arsenazo III colorimetric test [6] and ICP analysis of liquid after dialysis of the TMS. Further, atomic force microscopy (AFM) measurement of the TMS showed the majority of the nanoparticles are 1.1-1.3 nm in height with some particles approaching 3.0 nm to 4.0 nm, suggesting minimal agglomeration and/or aggregation.

The ATCA was developed as shown in Additional file 1 in which the TMS is functionalized with hydrophilic and lipophilic groups [4] and incorporated into liposomes [7]. The resulting liposome-TMS were formulated with or without CD36 ligands intercalated within the liposome bilayer membrane [5,8]. The ATCA was characterized as seen in Additional file 2.

### Cell culture

Foam cells were generated using oxidized-low density lipoproteins (oxLDL) from human plasma as described previously [9] and verified using oil red-o (ORO) staining [10]. The upregulation of CD36 expression in foam cells was confirmed using both PCR and FACs analysis with CD36-specific antibodies. As a control, the non CD36-expressing cell line 3 T3-F442A pre-adipocytes were used to detect non-specific binding. The U937 cells and foam cells were examined for toxicity using up to 100 μg/ml (five times the amount used for *in vivo* studies) for 10 days. No significant differences in cell viability was observed with ATCA-treated cells compared to controls (not shown) which further supports the observation that derivatized fullerenes are not toxic *in vitro*[11,12]. Cells were treated with CD36-targeted or non-targeted controls at various concentrations for 24 hrs, centrifuged and the supernatant saved for Gd analysis. After a quick wash the cell pellet was disrupted by sonication and supernatant and cell pellet subjected to ICP analysis or neutron bombardment (both methods gave similar results; not shown) to determine Gd concentration as described below.

### Cell uptake of the ATCA revealed by FIB-SEM crossbeam workstation

In order to examine cell uptake of the ATCA we used a Zeiss FIB-SEM crossbeam workstation. Foam cells were incubated with or without ATCA or non-targeted control (20 μg/ml) for various times (16, 36, 72 hrs). As a control non-targeted ATCA were incubated for the same times. In addition, non-foam cells were incubated with ATCA for 24 hrs. Cells were washed and fixed using 1% glutaraldehyde solution and 0.1 M cacodylate buffer for 2.5 hrs at room temperature. Samples were mounted on a microscope slide covered in aluminum foil and subjected to graded EtOH incubations (60%-100%/10 min each) and stored in a desiccated environment until microscopic analysis.

The mounted samples were put in the FIB-SEM crossbeam workstation, while a focused ion beam (FIB) was used to cut the cells and expose inner structure of the cell. Simultaneously, scanning electron microscope (SEM) was used to image the cross section milled via FIB, this is capable because both the ion beam and electron beam can focus on the exactly same area. The FIB was operated at 30 kV, 20 pA and SEM was operated at 1 kV to reduce potential beam charge and cell damage. A secondary electron detector was used to reveal morphology and energy. Additionally, the energy and angle selective backscattered electron (EsB) detector was applied to detect ATCA existence and spatial distribution with nanometer level resolution inside the cells, since heavy element Gd-containing C_80_ endohedrals will express themselves much brighter in EsB images if TMS exists.

### Western blotting and quantification of CD36-specific phospho-signaling intermediates

To confirm the specificity of CD36 ligand binding on the ATCA to the CD36 receptor and examine the mechanisms underlying the interactions between the ATCA and foam cells, Western blotting was used as described previously [13]. Following ATCA challenge, foam cells were lysed directly in boiling denaturing sample buffer consisting of tris-buffered saline with triton-X-100 (0.5%) and protease inhibitors. Proteins were separated on a 10% gels in tris-glycine SDS running buffer. Western blotting and image quantification was performed using the Odyssey Imaging System (LI-COR Biosciences). Band intensities were captured using the Odyssey scanner and bands quantified by measuring the number of pixels in each band using a box drawn for the same area of measurement for each separate blot. Band intensities were normalized for loading by dividing the number of pixels in each band with the housekeeping band intensity (β-actin) on the same blot.

### Flow cytometry

In order to confirm the upregulation of CD36 expression on foam cells, U937 cells (negative control) or PMA/oxLDL-transformed cells (foam cells) were washed with PBS/1% BSA, and blocked for 30 min at 4°C with a 1/500 dilution of normal human serum. The cells were washed and incubated with FITC-labeled anti-, CD36-FITC, or FITC-labeled isotype control (BD Biosciences; 10 μg/mL) for one hr. at 4°C. After three washes, cells were resuspended in 400 μL of PBS. The mean intensity of fluorescence was determined for at least 10,000 cells using a FACScan flow cytometer (BD Biosciences). All experiments were performed in duplicate.

### CMR study

The lesions in arteries of ApoE −/− mice progress from fatty streaks to foam cell-containing plaque in a similar way as humans [14,15]. ApoE −/− mice (23 weeks; female) and non-diseased controls were anesthetized initially with isoflurane (3%) and oxygen (3 L/min) in an induction chamber and kept under constant sedation via a nose cone while monitoring vital statistics as described [16]. Further details of the CMR are outlined in Additional file 3. CMR was performed blinded by personnel with no knowledge of targeted and non-targeted compounds. All animal studies were approved by Wake Forest School of Medicine Animal Care and Use Committee.

### Signal enhancement measurement

**Figure 1:**
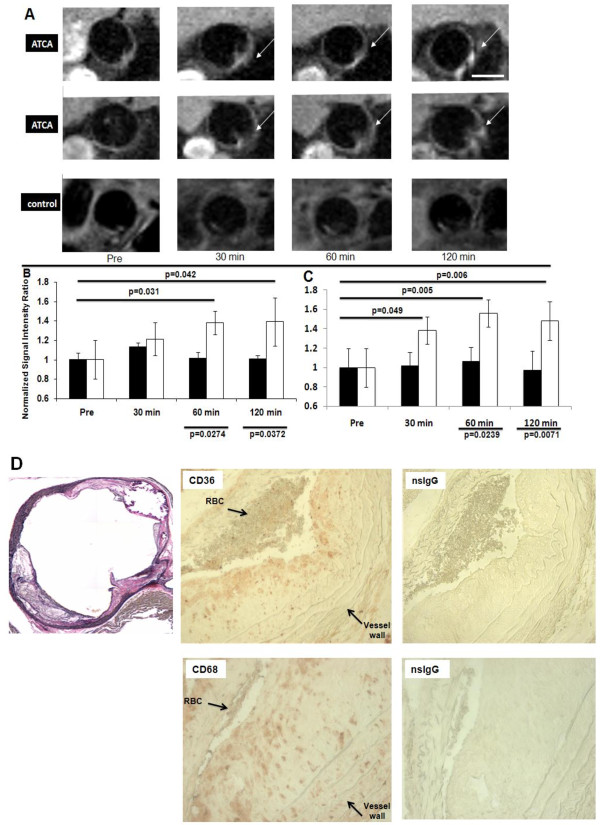
**ATCA can detect inflammatory plaque *in vivo. *** ApoE−/− mice (23 wks; n=5/grp) were injected i.v. (100 μg/100 μl) with ATCA (top and middle) or non-targeted control (bottom) and images acquired at the indicated times. Bar = 1.0 mm. Arrows indicate contrast agent binding to the plaque. **A**. Representative images from two (ATCA) or one (control) different mice injected with ATCA. **B**, **C**. Quantification of signal enhancement of ATCA. In **B**, the brightest voxels (approximately >10 spots/section) in the aorta wall from 5 separate animals were measured excluding artifact. The ratio of the Signal Intensity (SI) of the brightest voxel in the aorta wall to the SI of the (non-affected) myocardium for each time point was calculated. In **C**, the ratio of SI of the whole region lining the aorta wall to the SI of the (non-affected) myocardium was measured and the average signal intensity was recorded for 5 separate slices from each time-point. The SI-enhanced/SI-myo ratio from each time point to its pre scan SI-enhanced/SI-myo ratio were normalized (prescan values = 1). Data is expressed as mean of 5 CMR slices ± SD (n = 5; animals/grp; * indicates significance (p<0.05). **D**. Plaque lesions express macrophages and CD36 receptors. Aortas were stained with H&E (left), rat anti-mouse CD36 (middle), rat anti-mouse CD68 (bottom) or non-specific rat IgG (nsIgG). Peroxidase-conjugated anti-rat Abs were added and developed using AEC. The brown staining represents CD36- or CD68-positive cells located in the plaque lesions. Results are representative of at least three separate aortas.

Images acquired after the ATCA injection were examined for areas of hyper-intensity in the aorta wall (selected at random). As a comparison whole aortas were analyzed as described in Additional file 3. Regions appearing hyper-intense after ATCA injection were manually traced using ImageJ (nih.org). The contrast to noise ratio was calculated using a reference region of interest (ROI) next to the plaque lesion or of the whole aorta. The same regions were traced in the pre-ATCA injection image and in all the post-ATCA time point images. The contrast to noise was calculated using the same reference ROI and then the contrast-to-noise ratio (CNR) values were normalized to the pre-CA CNR value. The quantification of signal intensities were tabulated and analyzed for all variables and expressed as mean ± standard error (SE) from five separate animals per group (Figure 1B and Figure 1C). The StatPlus (Analyst Soft, 2011) software was used to evaluate a two (group) × two (time) analysis of variance (ANOVA) with repeated measures was evaluated to analyze data. Tukey LSD post hoc tests were used to examine pair-wise differences. Significance was set at p < 0.05.

### Histology, immunohistochemistry, and Gd detection in tissue

The aorta of each animal was removed and fixed in Tissue-Tek OCT (Miles, Elkhart, IN) and frozen with liquid nitrogen. The tissue was sectioned onto siloconized slides and histopathology assessed using hematoxylin for assessing plaque accumulation [17,18]. To examine CD36 and CD68 expression on macrophages in the plaque lesions the tissue sections were fixed in Carnoys, embedded in parafilm, and tissue sections placed on microscopic slides and probed with antibodies to CD36 and CD68 (both from Serotec) followed by horseradish-conjugated secondary antibodies and developed using AEC as described previously [19,20]. As a control non-specific, isotype-matched control antibodies (for CD36 and CD68) were used. Images were captured using a camera-equipped Zeiss AXIO Observer. For tissue distribution of Gd, organs (or ATCA sample used to inject to determine total Gd counts) were weighed and placed in pyrex tubes and combusted to ash at 900°C for 1 hr. The samples were again weighed and resuspended in 1.0 ml of 12 M (37%) HCl. The Gd was measured using ICP and percent Gd in tissue calculated based on the total amount of Gd in the ATCA sample used for injections.

### Toxicology evaluation

ApoE −/− were injected i.v. with PBS or 1,000 μg/100 μl (10 times more than optimized for imaging studies) of ATCA. Mice (four/group) were sacrificed at days two, seven, and 14 and alanine aminotranferease (ALT) and aspartate aminotransferase (AST) levels evaluated in serum. The ALT and AST are transaminase enzymes that leak out into the general circulation when the liver is injured. In separate experiments, ATCA were injected as above, tissue harvested at day seven, and subjected to neutron bombardment or ICP for Gd quantification. An aliquot of the ATCA (not injected) was measured separately to determine the percentage cleared from the mice.

## Results

### Plaque targeting contrast agents are selectively taken up by foam cells through CD36 receptor

**Figure 2:**
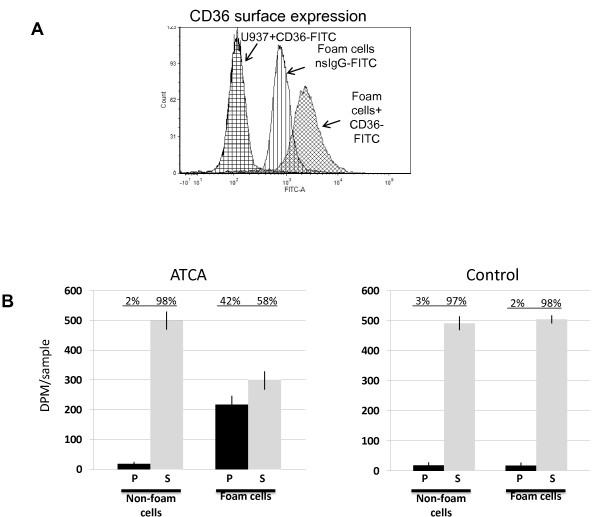
**A. Upregulation of CD36 receptors on foam cells.** U937 cells (in duplicate) were treated as described in Methods, washed and incubated with antibodies to CD36 or an isotype matched control. The graph shows the average mean fluorescent intensity (MFI) of the signal (±SD) and is representative of three separate experiments. **B**. Testing ATCA for foam cell binding. U937 cells (non-foam) were converted into foam cells (foam) using oxLDL and PMA as described. Cells were washed and ATCA (10 μg/ml) added overnight in a CO_2_ incubator. The next day cells were washed with media and the supernatants (S) and pellets (P) subjected to ICP for Gd detection. The percentage of Gd in the pellet and supernatant was calculated based on the total amount added to the cells.

Atherosclerotic plaque have higher numbers of macrophage foam cells that express CD36 scavenger receptors on their cell surface [21]. Given that the CD36 actively uptake extracellular lipids into their cytoplasmic membrane [22], we hypothesized that this may be a way to incorporate the ATCA contrast agent into foam cells in sufficient enough quantities that CMR could be performed. Foam cells were induced using PMA/oxLDL and the upregulation of CD36 expression was confirmed using FACs analysis (Figure 2A). As seen in Figure 2B, ATCA had a significant uptake into the CD36-expressing foam cells. The control compound for ATCA without the CD36 targeting moiety ligands (control) was not taken up within the cells suggesting the CD36 receptor was responsible for the uptake of the compounds within the cells. In data not shown, there was no uptake in non-foam cell monocytes or non-monocytic, tissue cells (3 T3).

**Figure 3:**
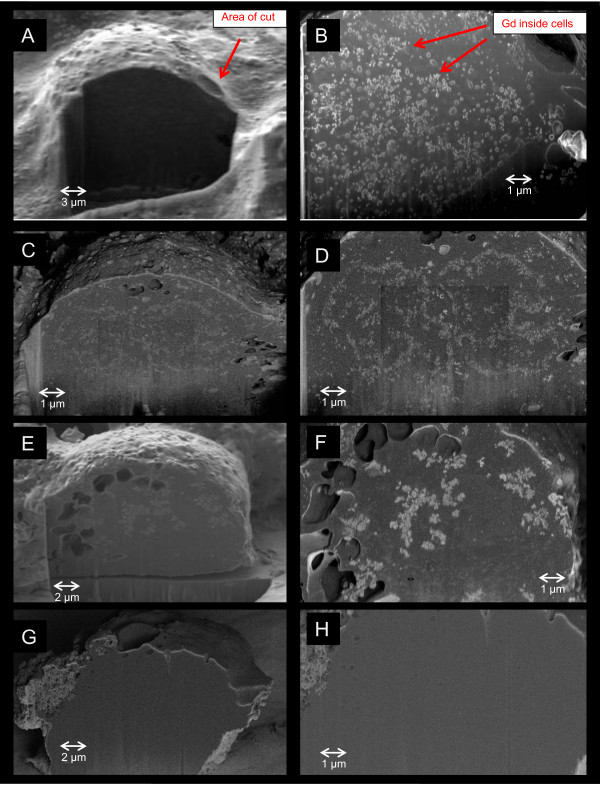
**A-H. ATCA are taken up by foam cells.** Foam cells were incubated with ATCA (10 μg/ml; **A**-**F**) or non-targeted control (**G**,**H**) for 16 (**A**,**B**), 48 (**C**,**D**) or 72 (**E**,**F**) hrs, washed, fixed, and subjected to FIB-SEM. Left columns (**A**,**C**,**E**,**G**) shows the morphology images taken by secondary electron detector after focused ion beam cut and the right column (**B**,**D**,**F**,**H**) shows the corresponding material contrast images taken by energy and angle selective backscattered electron (EsB) detector for the same region. The foam cell-targeted ATCA are clearly revealed inside the cells but not in the non-targeted controls. In parallel, an aliquot of cells were used to measure cell viability by trypan blue exclusion and revealed no cell death induced by ATCA addition compared to non-treated controls (not shown).

We next used a FIB-SEM - to follow the ATCA inside foam cells and determine the length of time it remains. Figure 3A-3H demonstrates that fullerene-based compounds can be detected inside human foam cells. The ATCA is shown to be more scattered inside the cell at earlier time points (B,D) and begins to aggregate with lesser amounts inside the cell at later time points (F) suggesting cell excretion. The non-targeted control was not detected inside the foam cells (G, H) further suggesting the specificity of the ATCA for foam cells.

**Figure 4:**
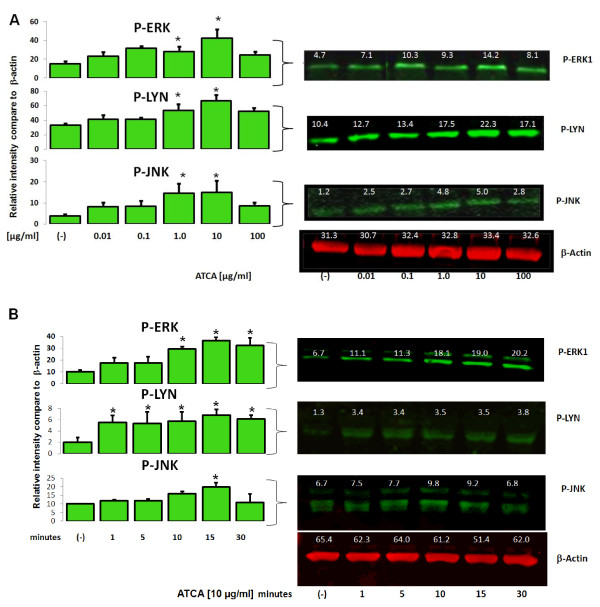
**A,B. ATCA activate CD36-specific signaling intermediates.** Foam cells were incubated with or without varying concentrations of ATCA (**A**; dose response) for 10 min or with 10 μg/ml ATCA (**B**; time course) for varying times. Cells were lysed and Western blotted using phospho-specific antibodies. Band intensities were quantified and the numbers represent the fold increase of each band compared to the housekeeping gene β-actin which did not demonstrate any change upon ATCA challenge. Results are representative of three separate experiments.

To definitively establish the specificity of ATCA binding to the CD36 receptor we used Western blotting and quantification of CD36-associated signaling molecules induced by ATCA treatment. Previous studies have shown that Erk, Lyn, and JNK2 are activated by the binding of oxLDL to CD36 receptors on macrophages [23,24]. When foam cells were challenged with ATCA there was a dose (Figure 4A) and time (Figure 4B) dependent activation of these signaling molecules. This provides definitive evidence that ATCA specifically target foam cells through CD36 receptors.

### ATCA detect atherosclerotic plaque *in vivo*

We used ATCA to visualize atherosclerotic disease in ApoE −/− mice [14]. As seen in Figure 1A (top and middle), mice injected i.v. with the ATCA had a striking enhanced T1 image of the plaque attached in the mouse aorta that could not be seen prior to injection. Of note is the observation that the imaging agent accumulates over time, suggesting it circulates through the blood for periods long enough for biomarker targeting to occur (Supplemental Video). Control compounds without the CD36 ligands demonstrated no accumulation in the vessel walls of ApoE −/− mice (Figure 1A-bottom). Quantification of the image intensity of ROI’s appearing after ATCA injection (Figure 1B) demonstrates accumulation of the compounds occurs after 30 min; one and two hour time-points demonstrated the optimal imaging time. When measuring the entire aorta the normalized mean intensity was very similar to what was observed in the bright spot ROI’s (Figure 1C). This quantification clearly demonstrates the ATCA significantly increases the contrast representing plaque lesions.

Separate experiments were performed with non-diseased animals. In these experiments the ATCA and control were injected and the descending aorta imaged at the same time points. As seen in the bottom panels wild-type C57 mice did not demonstrate any signal enhancement in the descending aorta further demonstrating the specificity of the ATCA for plaque detection. These results suggest the ATCA specifically detect disease-induced plaque lesions that are not expressed in normal vessel walls. Histochemical evaluation of each animal revealed that plaque lesions were present in the diseased animals (Figure 1D; left) with the accumulation of CD36- and CD68-positive cells on the anterior portion of the plaque lesions (Figure 1D; middle/bottom).

### Tracking the *in vivo* fate and toxicity of plaque-targeting contrast agents

**Figure 5:**
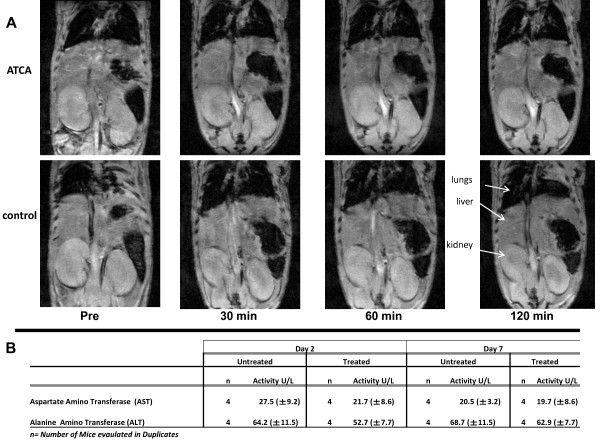
**A. Plaque-targeting contrast agents do not accumulate in major organs.** A representative plaque-targeting contrast agent and non-targeted control was injected as above and the mouse placed supine for whole body scan. Results are representative of four separate mice. **B**. **C**. ATCA does not accumulate in liver or induce toxicity. Mice were treated with or without 1000 μg/100 μl of ATCA via tail vein injection. Two and six days later the serum was used for ALT and AST levels. Data is presented as an average of four (untreated) or four (treated) mice ± SD. No increase in activity was observed between the untreated and treated samples. Activity is measure by Units/L obtained using linear regression from a standard curve.

In order to determine the fate of the ATCA we performed whole body scans after i.v. injection as described above. In all experiments the injection was well tolerated in each group of mice with no noticeable adverse reactions observed. As determined using CMR, there was no accumulation of any of the compounds in any major organs (e.g. liver and kidneys) at the same time points measured in the ApoE −/− mice (Figure 5A). To confirm these results we performed ICP analysis on the organs of these animals after seven days post injection. As seen in Table 1 Gd was only detected in the liver and kidneys demonstrating that approximately 85% of the ATCA is excreted from the body at this time-point.

**Table 1:** Total percent Gd in tissue

**Day 7 (n=4)**		
	**Average**	**SD**
Liver	9%	±6%
Heart	ND	ND
Spleen	ND	ND
Kidney	3%	±2%
Lung	ND	ND

Total Gd in tissue was valuate compared to total Gd injected ND-none detected.

In separate experiments in which high concentrations of ATCA were injected, there was no significant increase in serum activity of ALT and AST between the untreated and ATCA-treated animals indicating no liver toxicity (Figure 5B). Thus, the ATCA do not affect liver function and are mostly cleared from the body within five days after injection.

## Discussion

The results presented here demonstrate that atherosclerotic plaque can be detected *in vivo* using targeted, Gd-based molecular imaging nanomaterials and standard CMR techniques. Our rationale for developing ATCA was driven by the need for new ways to diagnose blood vessel plaque burden with existing technologies such as CMR. It is well established that atherosclerotic plaque express high levels of CD36 receptors for which ligands have already been identified. Importantly, these receptors normally and actively internalize the ligands (oxLDL); a prerequisite for accumulation of sufficient levels so that enhanced contrast can be observed. It is shown that this strategy was successful in identifying new ATCA which may provide new tools for physicians to detect plaque buildup in patients.

Magnetic resonance imaging is one of many techniques used to assess atherosclerotic plaque lesions in patients [25,26]. We functionalized the TMS through the addition of CD36 ligands which were hypothesized to home to macrophage foam cells found in atherosclerotic plaque [27-30]. Indeed, many studies have used this strategy of targeting diagnostics to macrophages through scavenger receptors as well as other macrophage-expressed biomarkers through several techniques including CMR [27,31-41]. For example, the Fayad group investigated the ability of macrophage scavenger receptor-targeted immunomicelles to assess macrophages in atherosclerotic plaque using CMR *in vivo*. The targeted micelles increased the signal intensity of atherosclerotic aortas 79% in ApoE−/− mice compared with only 34% using untargeted controls [27]. Others have targeted macrophages through the use of Lyp-1 peptides attached to a fluorescent dye [34]. Thus, targeting macrophages in atherosclerotic lesions is a valid strategy for developing diagnostics capable of detecting and quantifying plaque lesions. The advantages of using the CD36 receptor for targeting CMR contrast agents to plaque is its well established connection in atherosclerosis [28,30], its high expression on plaque foam cells [28], and its ability to be internalized upon ligand binding [42] which allows the accumulation of the ATCA ligand even under physiological flow conditions as was shown in Figure 1. The key limitation presently is the lack of a specific biomarker (or biomarkers) for plaque lesions that will not cross-react with other sites in the body. An even more difficult challenge is identifying vulnerable plaque biomarkers.

We used the Gd-containing TMS as they provide a unique starting material for developing biomarker-targeting diagnostics [43]. The TMS offers several advantages over current Gd-based CMR contrast agents for use as targeted diagnostics. First, the toxic Gd (in cage) is separated from active targeting moieties (outside cage) by the carbon shell. Adding targeting ligands/moieties to current contrast agents may affect the ability of Gd to become free of the compound. Of course, further studies are needed for these molecular entities but we and others have not observed any toxicity of empty cage or TMS (Figure 5) using several *in vitro* and *in vivo* assays [12,44,45]. Second, the TMS are more sensitive than existing contrast agents with three Gd/molecule compared to one Gd/molecule in commercially available chelates [43]. Targeted imaging agents require strong signals by which to report the presence of an agent at a particular location. Third, the fullerene cage can be targeted to disease biomarkers or encapsulated into liposomes/micelles without compromising release of Gd into the body; a strategy already being used for targeting glioblastoma [46]. The chemistry for the addition of different moieties to the cage offers many possibilities for not only biomarker targeting of plaque accumulation but also detection of biomarkers associated with removal of lesions (e.g. peptidoglycans) so that new tools for evaluative diagnosis of this disease can be developed.

The specificity of the ATCA for targeting foam cells through CD36 is demonstrated three separate ways. First, ICP analysis of Gd within foam cells was confirmed in Figure 2B demonstrating that the CD36 ligand-decorated ATCA were significantly taken up within foam cells while the non-targeted ATCA were not. Second, we used FIB-SEM; a powerful technology that can be used to cut the cell open and detect nanomaterials, to further show the targeted ATCA is specifically internalized within foam cells were levels begin to diminish after 72 hrs. This suggests the cells remain viable and can continue to exocytose as a normal macrophage would. Third, we show the targeted ATCA, and not the non-targeted, specifically activate signal transduction intermediates in the CD36 receptor pathway including the ligand binding-dependent phosphorylation of Erk, Lyn, and Jnk. Thus, the ATCA specifically detects and is taken up by macrophage foam cells though the CD36 receptor.

The use of ApoE −/− mice to study CMR contrast agents has been described extensively [14,15]. We demonstrate that the ATCA specifically recognize atherosclerotic plaque in diseased animals. No contrast was observed with non-targeted controls or in ATCA-treated, non-diseased animals. The signal enhancement occurs in a time-dependent fashion suggesting the foam cells are actively internalizing the ATCA via the CD36 scavenger receptor. While it has been reported that the lesion burden can vary among animals it was demonstrated that each animal tested had high levels of plaque lesions containing macrophages and CD36 receptors as identified using histochemistry and immunohistochemistry (Figure 1D). While each animal was verified to contain inflammation one limitation of this study was the small numbers of animals used. We are in the process of verifying these results in higher-order mammals (rabbits). The high power 7 T scanner used warrants further validation of the ATCA in a more clinically relevant scanner. However, the TMS have been studied in lower field magnets and demonstrate similar relaxivity and sensitivity [47,48].

Investigation of biocompatibility and biodistribution of the TMS-based protein cages *in vivo* is critical for future clinical translation. To this end, the studies examining the toxicity of fullerenes on human systems are still emerging and the subject of debate [44,49]. Several *in vivo* publications suggest that (in general) fullerenes show no overt signs of toxicity up to 2000 mg/kg [50,51] and have been shown to extend the lives (11%) and improve cognitive function of mice chronically treated with these compounds [52]. More recently, rats given fullerenes in olive oil lived over 90% longer than control mice [53]. We demonstrated that a single dose of ATCA, given at a 100 fold concentration higher than that needed for plaque imaging *in vivo*, caused no overt signs of toxicity, does not accumulate in the liver, and does not induce liver damage. These results are consistent with the observation that TMS injected i.v. into mice is passed through the kidney and into the urine (data not shown). The ATCA is mostly cleared from the body after seven days as demonstrated above. Similarly, the TMS were recently shown to have a favorable toxicity profile *in vitro* and *in vivo*[12]. Since it is anticipated that a patient will only be given a single injection before CMR is performed, these results are encouraging for future studies in humans. However, more studies are clearly needed to examine the long-term effects of fullerenes on biological systems. Efforts are focused on systematically identifying any acute and long-term toxicity issues with ATCA.

## Conclusions

This study describes the synthesis and testing of a new class of ATCA based on a novel metallofullerene nanomaterial. *In vitro* screening was used to select high relaxivity compounds that were taken up by foam cells via the scavenger receptor CD36. The CD36 compounds were shown to target inflammatory lesions in ApoE −/− mice while the same compounds without the CD36 ligands did not. These and other similar studies discussed above suggest the rationale for targeting macrophage foam cell may provide physicians with new tools for non-invasively diagnosing and quantifying otherwise undetectable plaque lesions. These results also suggest the TMS may serve as a platform to attach other biomarkers to target and image disease-specific biomarkers.

## Competing interests

All authors have declared no competing interests.

## Authors’ contributions

AD: substantial contributions to collection, analysis, and interpretation of data, and drafting/revision of the manuscript. JO: substantial contributions to acquisition and interpretation of MRI contrast data. KL: substantial contributions to acquisition and interpretation of MRI contrast data. SV: provided substantial contributions to interpretation and characterization of TMS. MS: provided substantial contributions to interpretation and characterization of TMS. JY: substantial contributions to acquisition and interpretation of microscopic data. ZZ: substantial contributions to design and synthesis of the atherosclerotic-targeting contrast agent, and revision of the manuscript. CK: substantial contributions to design of study, interpretation of data, and drafting/revision of manuscript. All authors read and approved the final manuscript.

## Supplementary Material

Additional file 1 Design and synthesis of ATCA. Click here for file

Additional file 2 A. DLS, zeta potential, and relaxivity of liposome control and ATCA. Click here for file

Additional file 3 MRI analysis. Click here for file

## References

[B1] Buxton DB Antman M Danthi N Report of the national heart, lung, and blood institute working group on the translation of cardiovascular molecular imaging Circulation 2011 123 19 2157 2163 10.1161/CIRCULATIONAHA.110.000943 21576680 PMC3119488

[B2] Underhill HR Hatsukami TS Fayad ZA Fuster V Yuan C MRI of carotid atherosclerosis: clinical implications and future directions Nat Rev Cardiol 2010 7 3 165 173 10.1038/nrcardio.2009.246 20101259

[B3] Stevenson S Fowler PW Heine T A stable non-classical metallofullerene family Nature 2000 408 6811 427 428 10.1038/35044199 11100715

[B4] MacFarland DK Walker KL Lenk RP Hydrochalarones: a novel endohedral metallofullerene platform for enhancing magnetic resonance imaging contrast J Med Chem 2008 51 13 3681 3683 10.1021/jm800521j 18558670 PMC2630425

[B5] Podrez EA Poliakov E Shen Z Identification of a novel family of oxidized phospholipids that serve as ligands for the macrophage scavenger receptor CD36 J Biol Chem 2002 277 41 38503 38516 10.1074/jbc.M203318200 12105195

[B6] Nagaraja TN Croxen RL Panda S Application of arsenazo III in the preparation and characterization of an albumin-linked, gadolinium-based macromolecular magnetic resonance contrast agent J Neurosci Methods 2006 157 2 238 245 10.1016/j.jneumeth.2006.05.013 16769125

[B7] Zhou Z Lenk RP Dellinger A Wilson SR Sadler R Kepley CL Liposomal formulation of amphiphilic fullerene antioxidants Bioconjug Chem 2010 21 9 1656 1661 10.1021/bc1001664 20839887 PMC2941224

[B8] Bligh EG Dyer WJ A rapid method of total lipid extraction and purification Can J Biochem Physiol 1959 37 8 911 917 13671378 10.1139/o59-099

[B9] Kuzuya M Yamada K Hayashi T Role of lipoprotein-copper complex in copper catalyzed-peroxidation of low-density lipoprotein Biochim Biophys Acta 1992 1123 3 334 341 10.1016/0005-2760(92)90015-N 1536873

[B10] Koopman R Schaart G Hesselink MK Optimisation of oil red O staining permits combination with immunofluorescence and automated quantification of lipids Histochem Cell Biol 2001 116 1 63 68 11479724 10.1007/s004180100297

[B11] Norton SK Dellinger A Zhou Z A new class of human mast cell and peripheral blood basophil stabilizers that differentially control allergic mediator release Clin Transl Sci 2010 3 4 158 169 10.1111/j.1752-8062.2010.00212.x 20718816 PMC5350695

[B12] Ehrich M Van Tassell R Li Y Zhou Z Kepley CL Fullerene antioxidants decrease organophosphate-induced acetylcholinesterase inhibition in vitro Toxicol In Vitro 2011 25 1 301 307 10.1016/j.tiv.2010.09.010 20888407

[B13] Tkaczyk C Metcalfe DD Gilfillan AM Determination of protein phosphorylation in Fc varepsilon RI-activated human mast cells by immunoblot analysis requires protein extraction under denaturing conditions J Immunol Methods 2002 268 2 239 243 10.1016/S0022-1759(02)00210-7 12215392

[B14] Kolovou G Anagnostopoulou K Mikhailidis DP Cokkinos DV Apolipoprotein E knockout models Curr Pharm Des 2008 14 4 338 351 10.2174/138161208783497769 18289060

[B15] Rosenfeld ME Averill MM Bennett BJ Schwartz SM Progression and disruption of advanced atherosclerotic plaques in murine models Curr Drug Targets 2008 9 3 210 216 10.2174/138945008783755575 18336239 PMC2942086

[B16] Wiant D Atwood TF Olson J Gamma knife radiosurgery treatment planning for small animals using high-resolution 7 T micro-magnetic resonance imaging Radiat Res 2009 172 5 625 631 10.1667/RR1614.1 19883231 PMC2800830

[B17] Alsaid H De SG Bourdillon MC Biomimetic MRI contrast agent for imaging of inflammation in atherosclerotic plaque of ApoE−/− mice: a pilot study Invest Radiol 2009 44 33 151 19169144 10.1097/RLI.0b013e31819472ac

[B18] Moukdar F Robidoux J Lyght O Pi J Daniel KW Collins S Reduced antioxidant capacity and diet-induced atherosclerosis in uncoupling protein-2-deficient mice J Lipid Res 2009 50 1 59 70 18698091 10.1194/jlr.M800273-JLR200

[B19] Anne-Marie I Candice H Han-Zhang X Immunohistochemical detection of human basophils in late-phase skin reactions J Allergy ClinImmunol 1997 101 3 354 362 10.1016/S0091-6749(98)70248-9 9525452

[B20] Kepley CL Craig SS Schwartz LB Identification and partial characterization of a unique marker for human basophils J Immunol 1995 154 12 6548 6555 7759888

[B21] Nicholson AC Han J Febbraio M Silversterin RL Hajjar DP Role of CD36, the macrophage class B scavenger receptor, in atherosclerosis Ann NY Acad Sci 2001 947 224 228 11795270 10.1111/j.1749-6632.2001.tb03944.x

[B22] Brown MS Basu SK Falck JR Ho YK Goldstein JL The scavenger cell pathway for lipoprotein degradation specificity of the binding site that mediates the uptake of negatively-charged LDL by macrophages J Supramol Struct 1980 13 1 67 81 10.1002/jss.400130107 6255257

[B23] Collins RF Touret N Kuwata H Tandon NN Grinstein S Trimble WS Uptake of oxidized low density lipoprotein by CD36 occurs by an actin-dependent pathway distinct from macropinocytosis J Biol Chem 2009 284 44 30288 30297 10.1074/jbc.M109.045104 19740737 PMC2781584

[B24] Rahaman SO Lennon DJ Febbraio M Podrez EA Hazen SL Silverstein RL A CD36-dependent signaling cascade is necessary for macrophage foam cell formation Cell Metab 2006 4 3 211 221 10.1016/j.cmet.2006.06.007 16950138 PMC1855263

[B25] Partovi S Loebe M Aschwanden M Contrast-enhanced ultrasound for assessing carotid atherosclerotic plaque lesions AJR Am J Roentgenol 2012 198 1 W13 19 10.2214/AJR.11.7312 22194509

[B26] Voros S Rinehart S Qian Z Coronary atherosclerosis imaging by coronary CT angiography: current status, correlation with intravascular interrogation and meta-analysis JACC Cardiovasc Imaging 2011 4 5 537 548 10.1016/j.jcmg.2011.03.006 21565743

[B27] Amirbekian V Lipinski MJ Briley-Saebo KC Detecting and assessing macrophages in vivo to evaluate atherosclerosis noninvasively using molecular MRI Proc Natl Acad Sci USA 2007 104 3 961 966 10.1073/pnas.0606281104 17215360 PMC1766334

[B28] Collot-Teixeira S Martin J rmott-Roe C Poston R McGregor JL CD36 And macrophages in atherosclerosis Cardiovasc Res 2007 75 3 468 477 10.1016/j.cardiores.2007.03.010 17442283

[B29] Kahn E Vejux A Menetrier F Analysis of CD36 expression on human monocytic cells and atherosclerotic tissue sections with quantum dots: investigation by flow cytometry and spectral imaging microscopy Anal Quant Cytol Histol 2006 28 1 14 26 16566276

[B30] Nergiz-Unal R Rademakers T Cosemans JM Heemskerk JW CD36 As a multiple-ligand signaling receptor in atherothrombosis Cardiovasc Hematol Agents Med Chem 2011 9 1 42 55 10.2174/187152511794182855 20939828

[B31] Lipinski MJ Amirbekian V Frias JC MRI to detect atherosclerosis with gadolinium-containing immunomicelles targeting the macrophage scavenger receptor Magn Reson Med 2006 56 3 601 610 10.1002/mrm.20995 16902977

[B32] Ouimet T Lancelot E Hyafil F Molecular and cellular targets of the MRI contrast agent p947 for atherosclerosis imaging Mol Pharm 2012 9 4 850 861 10.1021/mp2003863 22352457

[B33] Lipinski MJ Frias JC Amirbekian V Macrophage-specific lipid-based nanoparticles improve cardiac magnetic resonance detection and characterization of human atherosclerosis JACC Cardiovasc Imaging 2009 2 5 637 647 10.1016/j.jcmg.2008.08.009 19442953 PMC2756539

[B34] Uchida M Kosuge H Terashima M Protein cage nanoparticles bearing the LyP-1 peptide for enhanced imaging of macrophage-rich vascular lesions ACS Nano 2011 5 4 2493 2502 10.1021/nn102863y 21391720 PMC3082619

[B35] Hyafil F Cornily JC Feig JE Noninvasive detection of macrophages using a nanoparticulate contrast agent for computed tomography Nat Med 2007 13 5 636 641 10.1038/nm1571 17417649

[B36] Davies JR Rudd JH Weissberg PL Narula J Radionuclide imaging for the detection of inflammation in vulnerable plaques J Am Coll Cardiol 2006 47 8 Suppl C57 68 16631511 10.1016/j.jacc.2005.11.049

[B37] Nahrendorf M Jaffer FA Kelly KA Noninvasive vascular cell adhesion molecule-1 imaging identifies inflammatory activation of cells in atherosclerosis Circulation 2006 114 14 1504 1511 10.1161/CIRCULATIONAHA.106.646380 17000904

[B38] Nahrendorf M Sosnovik DE Weissleder R MR-optical imaging of cardiovascular molecular targets Basic Res Cardiol 2008 103 2 87 94 10.1007/s00395-008-0707-2 18324364 PMC2664635

[B39] Rudd JH Warburton EA Fryer TD Imaging atherosclerotic plaque inflammation with [18 F]-fluorodeoxyglucose positron emission tomography Circulation 2002 105 23 2708 2711 10.1161/01.CIR.0000020548.60110.76 12057982

[B40] Kooi ME Cappendijk VC Cleutjens KB Accumulation of ultrasmall superparamagnetic particles of iron oxide in human atherosclerotic plaques can be detected by in vivo magnetic resonance imaging Circulation 2003 107 19 2453 2458 10.1161/01.CIR.0000068315.98705.CC 12719280

[B41] Canet-Soulas E Letourneur D Biomarkers of atherosclerosis and the potential of MRI for the diagnosis of vulnerable plaque MAGMA 2007 20 3 129 142 10.1007/s10334-007-0078-y 17605060

[B42] Endemann G Stanton LW Madden KS Bryant CM White RT Protter AA CD36 Is a receptor for oxidized low density lipoprotein J Biol Chem 1993 268 16 11811 11816 7685021

[B43] Fatouros PP Corwin FD Chen ZJ In vitro and in vivo imaging studies of a new endohedral metallofullerene nanoparticle Radiology 2006 240 3 756 764 10.1148/radiol.2403051341 16837672

[B44] Kolosnjaj J Szwarc H Moussa F Toxicity studies of fullerenes and derivatives Adv Exp Med Biol 2007 620 168 180 10.1007/978-0-387-76713-0_13 18217343

[B45] Norton SK Wijesinghe DS Dellinger A Epoxyeicosatrienoic acids are involved in the C(70) fullerene derivative-induced control of allergic asthma J Allergy Clin Immunol 2012 130 3 761 769 10.1016/j.jaci.2012.04.023 22664166 PMC3955256

[B46] Fillmore HL Shultz MD Henderson SC Conjugation of functionalized gadolinium metallofullerenes with IL-13 peptides for targeting and imaging glial tumors Nanomedicine (Lond) 2011 6 3 449 458 10.2217/nnm.10.134 21542684

[B47] Shu C Corwin FD Zhang J Facile preparation of a new gadofullerene-based magnetic resonance imaging contrast agent with high 1H relaxivity Bioconjug Chem 2009 20 6 1186 1193 10.1021/bc900051d 19445504 PMC2862651

[B48] Zhang J Fatouros PP Shu C High relaxivity trimetallic nitride (Gd(3)N) metallofullerene MRI contrast agents with optimized functionality Bioconjug Chem 2010 21 4 610 10.1021/bc900375n 20218678 PMC2862638

[B49] Aschberger K Johnston HJ Stone V Review of fullerene toxicity and exposure - appraisal of a human health risk assessment, based on open literature Regul Toxicol Pharmacol 2010 58 3 455 473 10.1016/j.yrtph.2010.08.017 20800639

[B50] Mori T Takada H Ito S Matsubayashi K Miwa N Sawaguchi T Preclinical studies on safety of fullerene upon acute oral administration and evaluation for no mutagenesis Toxicology 2006 225 1 48 54 10.1016/j.tox.2006.05.001 16782258

[B51] Gharbi N Pressac M Hadchouel M Szwarc H Wilson SR Moussa F [60]Fullerene is a powerful antioxidant in vivo with no acute or subacute toxicity Nano Lett 2005 5 12 2578 2585 10.1021/nl051866b 16351219

[B52] Quick KL Ali SS Arch R Xiong C Wozniak D Dugan LL A carboxyfullerene SOD mimetic improves cognition and extends the lifespan of mice Neurobiol Aging 2008 29 1 117 128 10.1016/j.neurobiolaging.2006.09.014 17079053

[B53] Baati T Bourasset F Gharbi N The prolongation of the lifespan of rats by repeated oral administration of [60]fullerene Biomaterials 2012 33 19 4936 4946 10.1016/j.biomaterials.2012.03.036 22498298

