# Supplementary material for: Functionalization of gadolinium metallofullerenes for detecting atherosclerotic plaque lesions by cardiovascular magnetic resonance
Source: J Cardiovasc Magn Reson. 2013 Jan 16;15(1):7. doi: 10.1186/1532-429X-15-7 (PMC3562260; doi:10.1186/1532-429X-15-7)
Supplement: Additional file 1 — Design and synthesis of ATCA. [file 1532-429X-15-7-S1.pptx]

## Slide 1
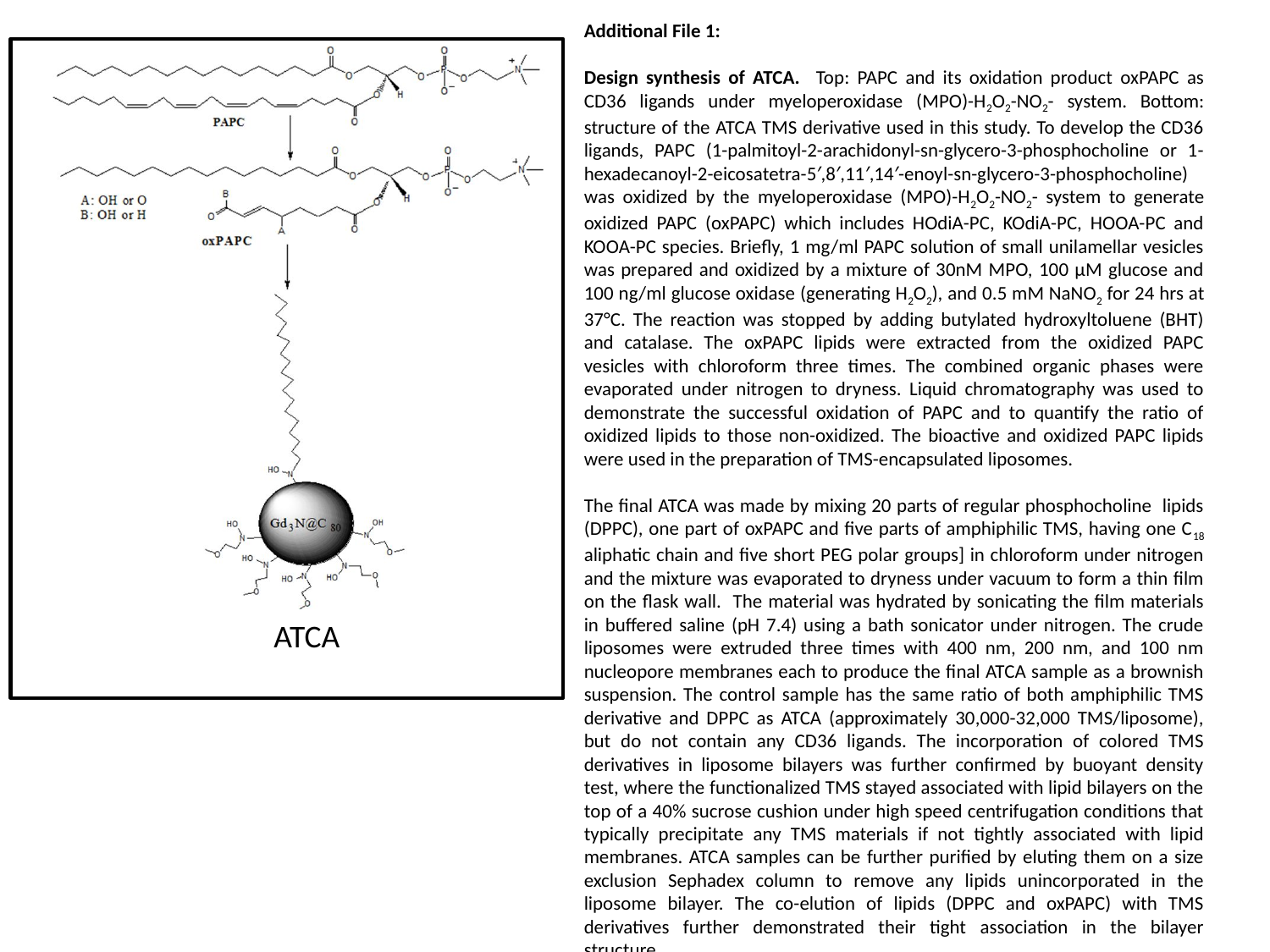

Additional File 1:
Design synthesis of ATCA. Top: PAPC and its oxidation product oxPAPC as CD36 ligands under myeloperoxidase (MPO)-H2O2-NO2- system. Bottom: structure of the ATCA TMS derivative used in this study. To develop the CD36 ligands, PAPC (1-palmitoyl-2-arachidonyl-sn-glycero-3-phosphocholine or 1-hexadecanoyl-2-eicosatetra-5′,8′,11′,14′-enoyl-sn-glycero-3-phosphocholine) was oxidized by the myeloperoxidase (MPO)-H2O2-NO2- system to generate oxidized PAPC (oxPAPC) which includes HOdiA-PC, KOdiA-PC, HOOA-PC and KOOA-PC species. Briefly, 1 mg/ml PAPC solution of small unilamellar vesicles was prepared and oxidized by a mixture of 30nM MPO, 100 μM glucose and 100 ng/ml glucose oxidase (generating H2O2), and 0.5 mM NaNO2 for 24 hrs at 37°C. The reaction was stopped by adding butylated hydroxyltoluene (BHT) and catalase. The oxPAPC lipids were extracted from the oxidized PAPC vesicles with chloroform three times. The combined organic phases were evaporated under nitrogen to dryness. Liquid chromatography was used to demonstrate the successful oxidation of PAPC and to quantify the ratio of oxidized lipids to those non-oxidized. The bioactive and oxidized PAPC lipids were used in the preparation of TMS-encapsulated liposomes.
The final ATCA was made by mixing 20 parts of regular phosphocholine lipids (DPPC), one part of oxPAPC and five parts of amphiphilic TMS, having one C18 aliphatic chain and five short PEG polar groups] in chloroform under nitrogen and the mixture was evaporated to dryness under vacuum to form a thin film on the flask wall. The material was hydrated by sonicating the film materials in buffered saline (pH 7.4) using a bath sonicator under nitrogen. The crude liposomes were extruded three times with 400 nm, 200 nm, and 100 nm nucleopore membranes each to produce the final ATCA sample as a brownish suspension. The control sample has the same ratio of both amphiphilic TMS derivative and DPPC as ATCA (approximately 30,000-32,000 TMS/liposome), but do not contain any CD36 ligands. The incorporation of colored TMS derivatives in liposome bilayers was further confirmed by buoyant density test, where the functionalized TMS stayed associated with lipid bilayers on the top of a 40% sucrose cushion under high speed centrifugation conditions that typically precipitate any TMS materials if not tightly associated with lipid membranes. ATCA samples can be further purified by eluting them on a size exclusion Sephadex column to remove any lipids unincorporated in the liposome bilayer. The co-elution of lipids (DPPC and oxPAPC) with TMS derivatives further demonstrated their tight association in the bilayer structure.
ATCA
