# Supplementary material for: Functionalization of gadolinium metallofullerenes for detecting atherosclerotic plaque lesions by cardiovascular magnetic resonance
Source: J Cardiovasc Magn Reson. 2013 Jan 16;15(1):7. doi: 10.1186/1532-429X-15-7 (PMC3562260; doi:10.1186/1532-429X-15-7)
Supplement: Additional file 2 — A. DLS, zeta potential, and relaxivity of liposome control and ATCA. [file 1532-429X-15-7-S2.pptx]

## Slide 1
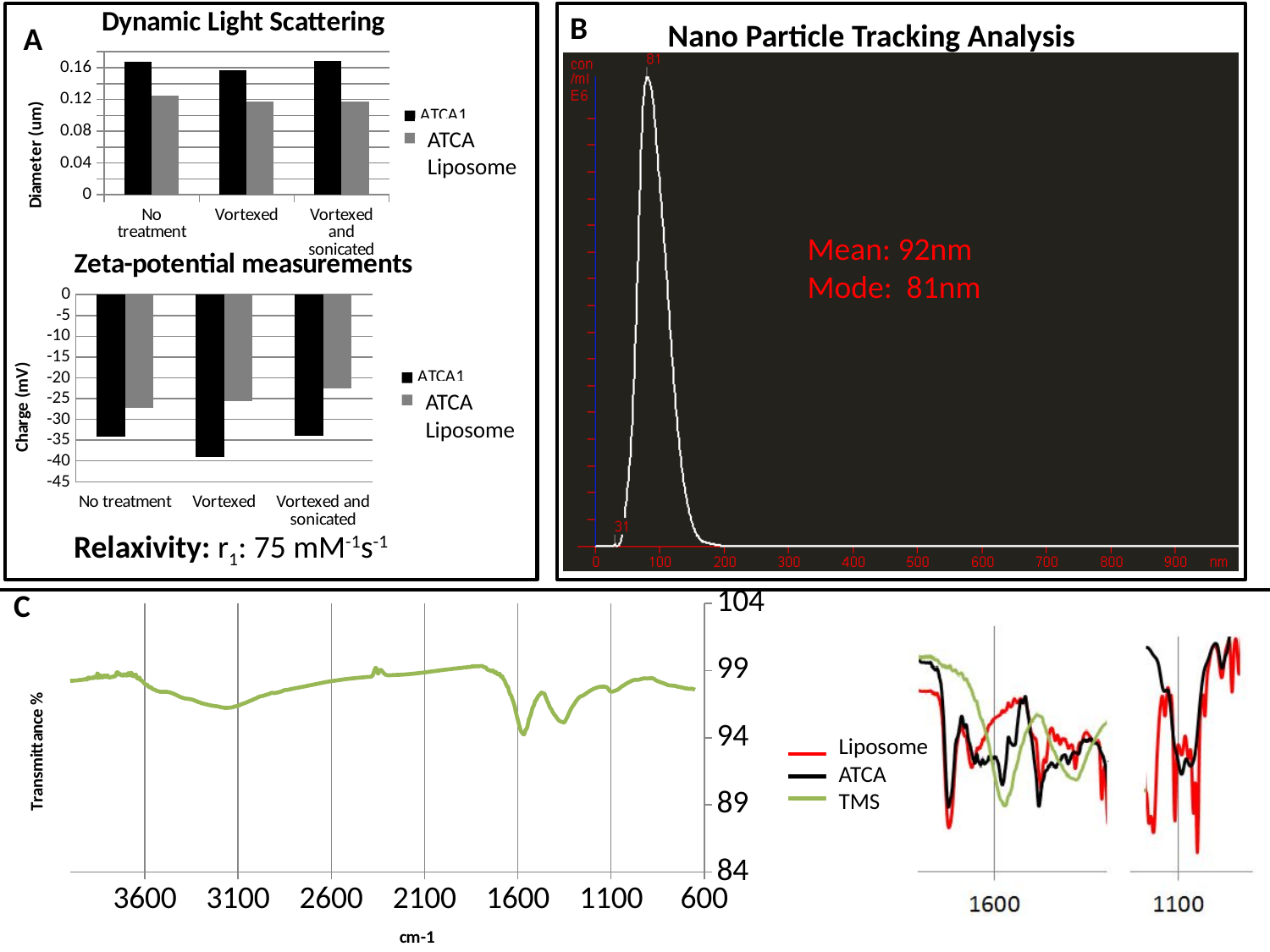

### Chart: Dynamic Light Scattering
| Category | ATCA1 | control |
|---|---|---|
| No treatment | 0.1672 | 0.1251 |
| Vortexed | 0.1565 | 0.1172 |
| Vortexed and sonicated | 0.1683 | 0.1178 |B
Nano Particle Tracking Analysis
A
ATCA
Liposome
Mean: 92nm
Mode: 81nm
### Chart: Zeta-potential measurements
| Category | ATCA1 | |
|---|---|---|
| No treatment | -34.19 | -27.3 |
| Vortexed | -39.02 | -25.5 |
| Vortexed and sonicated | -33.92 | -22.5 |ATCA
Liposome
C
### Chart
| Category | | | |
|---|---|---|---|
Liposome
ATCA
TMS
Relaxivity: r1: 75 mM-1s-1

## Slide 2
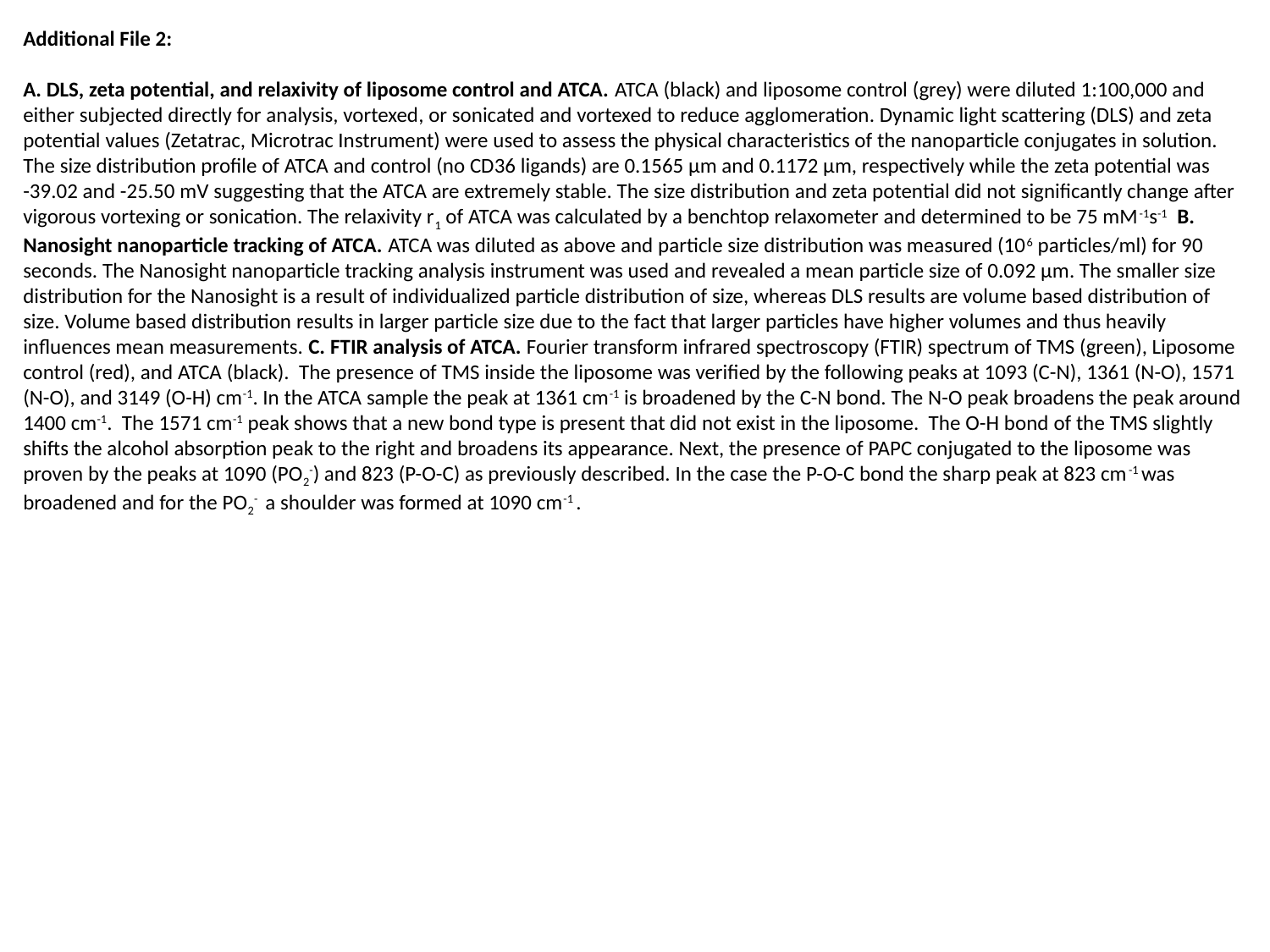

Additional File 2:
A. DLS, zeta potential, and relaxivity of liposome control and ATCA. ATCA (black) and liposome control (grey) were diluted 1:100,000 and either subjected directly for analysis, vortexed, or sonicated and vortexed to reduce agglomeration. Dynamic light scattering (DLS) and zeta potential values (Zetatrac, Microtrac Instrument) were used to assess the physical characteristics of the nanoparticle conjugates in solution. The size distribution profile of ATCA and control (no CD36 ligands) are 0.1565 μm and 0.1172 μm, respectively while the zeta potential was -39.02 and -25.50 mV suggesting that the ATCA are extremely stable. The size distribution and zeta potential did not significantly change after vigorous vortexing or sonication. The relaxivity r1 of ATCA was calculated by a benchtop relaxometer and determined to be 75 mM-1s-1 B. Nanosight nanoparticle tracking of ATCA. ATCA was diluted as above and particle size distribution was measured (106 particles/ml) for 90 seconds. The Nanosight nanoparticle tracking analysis instrument was used and revealed a mean particle size of 0.092 µm. The smaller size distribution for the Nanosight is a result of individualized particle distribution of size, whereas DLS results are volume based distribution of size. Volume based distribution results in larger particle size due to the fact that larger particles have higher volumes and thus heavily influences mean measurements. C. FTIR analysis of ATCA. Fourier transform infrared spectroscopy (FTIR) spectrum of TMS (green), Liposome control (red), and ATCA (black). The presence of TMS inside the liposome was verified by the following peaks at 1093 (C-N), 1361 (N-O), 1571 (N-O), and 3149 (O-H) cm-1. In the ATCA sample the peak at 1361 cm-1 is broadened by the C-N bond. The N-O peak broadens the peak around 1400 cm-1. The 1571 cm-1 peak shows that a new bond type is present that did not exist in the liposome. The O-H bond of the TMS slightly shifts the alcohol absorption peak to the right and broadens its appearance. Next, the presence of PAPC conjugated to the liposome was proven by the peaks at 1090 (PO2-) and 823 (P-O-C) as previously described. In the case the P-O-C bond the sharp peak at 823 cm-1 was broadened and for the PO2- a shoulder was formed at 1090 cm-1 .
