# Supplementary material for: Functionalization of gadolinium metallofullerenes for detecting atherosclerotic plaque lesions by cardiovascular magnetic resonance
Source: J Cardiovasc Magn Reson. 2013 Jan 16;15(1):7. doi: 10.1186/1532-429X-15-7 (PMC3562260; doi:10.1186/1532-429X-15-7)
Supplement: Additional file 3 — MRI analysis. [file 1532-429X-15-7-S3.docx]

**MRI analysis**

All MR Imaging experiments were performed on a Bruker 70/30 7T microMRI scanner equipped with a 60 mm I.D. 1000 mT/mm gradient and shim coil. MR signal transmission and reception was performed with a quadrature 35 mm I.D. volume Radio Frequency (RF) coil (magnet, gradient/shim coil, and RF coil are all from Bruker, Billerica, MA). Diseased mice or non-diseased age matched controls (C57/B6) 23 week old were group housed. The mice were fed Prolab RMH 3000 5P00 chow ad libitum. Chemical composition of the chow included: 22.5% protein, 5.4% fat (ether extract), 6.4% Fat (acid hydrolysis), and 4% fiber (crude).

Anesthesia was induced by placing the mouse in an induction chamber initially filled with room air, then flowing a mixture of isoflurane (3%) and O_2_ (3 L/min) into the induction chamber. When the animal was anesthetized, it was moved from the induction chamber into the magnet room, where it was placed in a mouse bed fitted with a nose cone that continued to supply isoflurne (1.5%) and O2 (1 L/min) to maintain anesthesia during scanning. The mouse was placed in a supine position so that its paws were accessible for the application of ECG leads. Neonatal ECG leads (3M, St. Paul MN) were attached to the mouse’s front right paw and left rear paw. The leads were connected to an ECG/T module that was interfaced with a PC running small animal monitoring software (SAII-Instruments, Stony Brook, NY). A respiration pillow was placed over the abdomen to monitor the respiration rate. The animal’s vital signs were monitored and isoflurane levels were adjusted to keep the respiration rate between 35 and 45 breaths per minute. The ECG wave form and respiration wave form also facilitated gated acquisition of MR data to reduce motion artifacts that are caused by cardiac and respiratory motion. The animal’s body temperature was monitored and maintained by blowing thermostatically controlled warm air into the bore of the magnet during the scan.

When the animal was anesthetized and connected to vital sign monitoring equipment, the mouse bed with the mouse secured in it was placed in the RF coil so that the aortic arch was at the iso center of the RF coil. The RF coil was tuned and matched to 300.2 MHz. Then a three plane localizer scout scan was acquired to verify that the mouse’s aortic arch was centered in the RF coil and in the magnet. An ungated Rapid Acquisition with Relaxation Enhancement (RARE) pulse sequence with an echo train length of eight was used for the three plane scout. Parameters for the RARE scans were: Repetition Time (TR) = 1500 ms, Echo Time (TE) = 25 ms, Field of View (FOV) =3.0 cm, Matrix = 128x128, slice thickness (thk) = 2.0 mm Number of Averages (NEX) = 1. If necessary, the mouse was repositioned in the RF coil or the RF coil was repositioned in the magnet or both. A follow up three plane scout scan was acquired to confirm that the mouse’s aortic arch was centered. When the mouse was in position, a series of ECG and respiratory gated low resolution Fast Low Angle Shot (FLASH) scout scans were acquired to facilitate the slice plane positioning for the high resolution. Parameters of the FLASH scout scans were: TR/TE/Flip Angle(FA)/FOV/Matrix/thk/NEX = 44ms/5ms/300/3.0cm/128x128/1.5mm/1. All gated scans were triggered when an R-wave peak was detected that fell in between breaths. The maximum width of the breath trigger window was monitored throughout scanning and was adjusted so that it started 100 ms after each breath and ended sooner than 400 ms before the next breath. Low resolution scout scans and the high resolution aorta images were acquired using a saturation slice and a fat suppression prepulse. The saturation slice was positioned to cover the ventricles to saturate the blood that would be flowing through the image plane during acquisition to suppress signal from flowing blood. The fat suppression pre-pulse was used to reduce chemical shift artifact that can be caused by fat surrounding the heart. The FLASH scout scans were acquired to generate an oblique coronal view of the aortic arch that clearly showed the three branches that exit the top of the aortic arch. This scout scan was used to place the high resolution ECG and respiratory gated FLASH sequence slice planes so that they covered the 3 mm proximal to the brachiocephallic artery branch point. The parameters for the high resolution FLASH sequence were: TR/TE/FA/FOV/Matrix/thk/NEX= 120ms/4.9ms/300/3.0cm/256x256/0.38mm/4 (giving a pixel size of 117 µm).

Experiments were performed using two personnel. The first injected either control or ATCA view tail vein. The second, blinded to the injections, performed the MRI scans and analysis. After the baseline high resolution scan was acquired, mice were injected with 100uL of ATCA (100ug) via tail vein. This concentration was determined to be optimal in initial experiments (not shown). The animal was placed back into the RF coil, the RF coil was placed back in the magnet and the protocol to locate the aortic arch and brachiocephalic artery was performed again to allow the acquisition of images at 30 minutes, 60 minutes, and 120 minutes post injection. The use of the brachiocephalic artery branch point as an anatomical reference allowed reproducible slice placement for the pre injection scan and the first post injection scan. All the post injection scans were acquired with the mouse and slice planes in the same position so they were the same for the post injection time points.

Animals were scanned at 30 minutes, 60 minutes, and 120 minutes post injection. The post injection scans were performed without moving the mouse so the animal position and slice positions for all the post injection scans were the same for each animal. The animals were euthanized using CO_2_ and cervical dislocation following scanning. The aortas were removed for analysis.

For quantification of the ATCA signal in the aorta two separate methods were used. First, the ROI of the brightest spots in the aorta walls were quantified (Figure 4B) using Image J Software. As a comparison, each reviewer (three) blinded to the compound being measured, quantified whole aorta walls from 4 separate 0.3 mm^2^ slices (slices 2-5 as slices 1 and 6 are too narrow) manually drawing the whole aorta wall and normalized to the surrounding muscle (same area measured). One reviewer drew the areas to be measured, the second reviewer read out the quantifiable numbers (See Table below), and the third reviewer recorded the numbers. When measuring the entire ROI of each slice (four ) at each time point (four) the mean intensity normalized to thorasic muscle is very similar to what was measured originally (Figure 4C)-the brightest ROI’s that was presented in Figure 4B. Both approaches equally quantify the significant enhanced contrast using ATCA. Statistical analysis is shown in the text and each experiment represents five separate animals.
